# Retraction: TRIF/miR-34a mediates aldosterone-induced cardiac inflammation and remodeling

**DOI:** 10.1042/CS-20200249_RET

**Published:** 2021-01-11

**Keywords:** aldosterone, cardiac remodeling, inflammation, microRNA-34a, TRIF

This Retraction follows an Expression of Concern relating to this article previously published by Portland Press.

The original article “TRIF/miR-34a mediates aldosterone-induced cardiac inflammation and remodeling” (*Clinical Science* (2020) **134**(12) DOI: 10.1042/CS20200249) is being retracted from *Clinical Science* at the request of the Editorial Board following receipt of a notification from a reader, raising concern regarding a suspected splice in Figure 1B. The authors are unable to provide the required original data to support a conclusive investigation and therefore the Editor-in-Chief and Editorial Board have retracted this article.

